# Supplementary material for: Non-human primates can flexibly learn serial sequences and reorder context-dependent object sequences
Source: PLoS Biol. 2025 Jun 23;23(6):e3003255. doi: 10.1371/journal.pbio.3003255 (PMC12208462; doi:10.1371/journal.pbio.3003255)
Supplement: S1 Fig — (A) Prop. correct choices for each ordinal position (y-axis) and trial (x-axis) for each of the four subjects for novel sequences in context 1. (B) Probability to reach 80% correct choices (y-axis) for each ordinal position (diff. colors) across trials (symbols mark different subjects). (C) Avg trial position that subjects completed in >80% of sequences (y-axis) for each ordinal position (diff. colors). Error bar indicates mean and 95% CI. Ordinal position 1: 1.03 trials (±0.03), 2: 1.27 (±0.09), 3: 1.85 (±0.19), 4: 2.62 (±0.32), and 5: 3.66 (±0.43). (D) Exploration errors, distractor errors (choosing the distractor object that was shown as a sequence-irrelevant sixth object in the display), and rule-breaking errors decreased over trials. Data were fit with an exponential decay function (y= a*exp(−bx)+c). (E) Mean (and 95% CI) of the decay factor (b) for each error type: Exploration error (0.14 [0.12, 0.17]), Rule breaking error (0.0041 [0.0025, 0.0058]), Distractor error (0.15 [0.12, 0.19]), and Perseverative error (0.022 [0.014, 0.029]). Exploration and distractor errors had a similar decay rate (p = 0.66). Stars denote significance levels for pairwise comparisons (Welch’s t-tests). Decay factors for all error types were significantly greater than zero, confirming a decrease in all error types over the course of learning (t test against zero, p-values: Exploration error: 6.6 × 10^−18^; Rule breaking error: 5.4 × 10^−6^; Distractor error: 7.3 × 10^−16^; Perseverative error: 4.3 × 10^−8^). The data underlying this figure can be found in the S1 Data file. (DOCX) [file pbio.3003255.s001.docx]

**Overall learning performance**

**
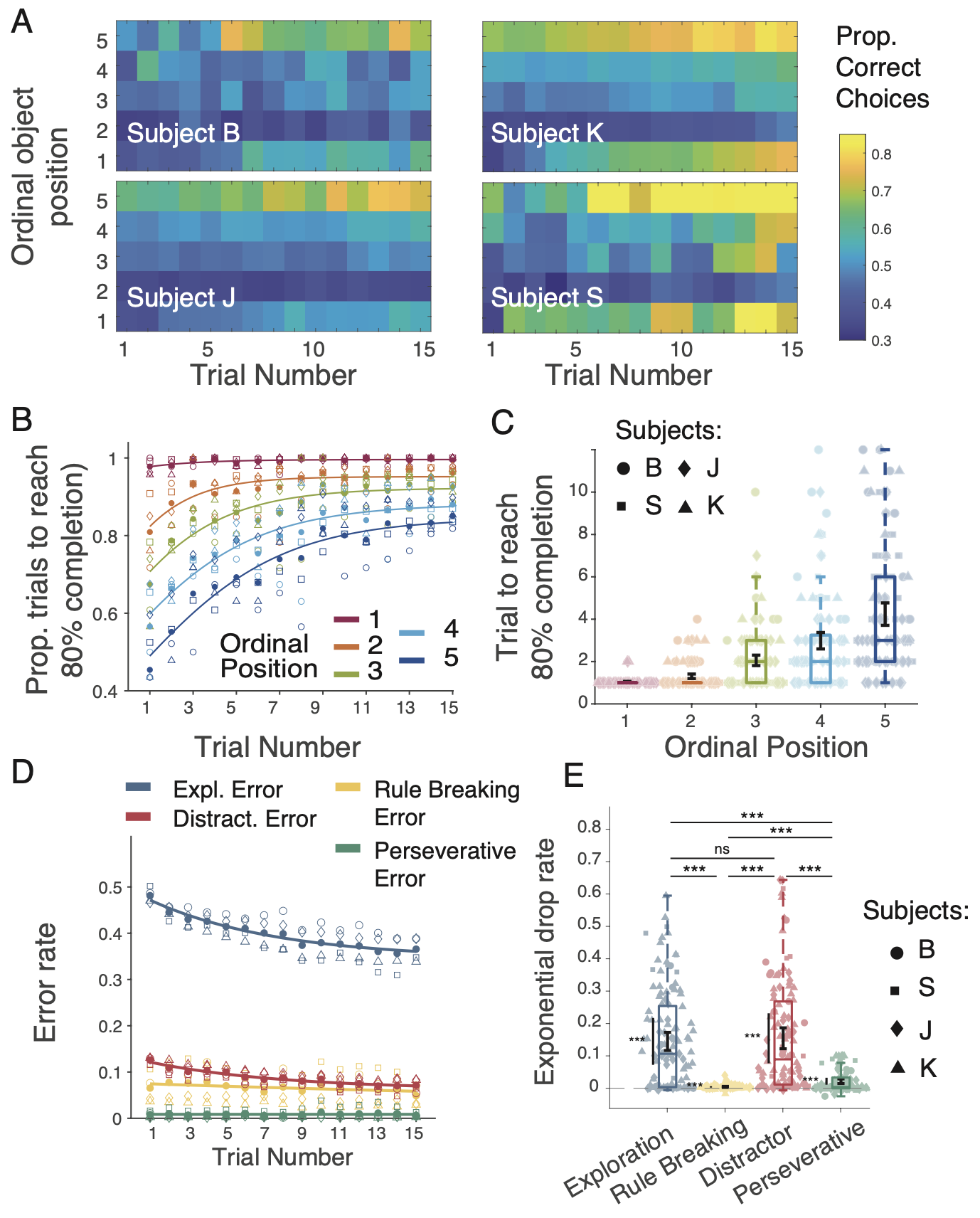
**

**S1 Fig. Overall learning performance.** (**A**) Prop. correct choices for each ordinal position (y-axis) and trial (x-axis) for each of the four subjects for novel sequences in context 1. (**B**) Probability to reach 80% correct choices (*y-axis*) for each ordinal position (diff. colors) across trials (symbols mark different subjects). (**C**) Avg trial position that subjects completed in >80% of sequences (y-axis) for each ordinal position (diff. colors). Error bar indicates mean and 95% CI. Ordinal position 1: 1.03 trials (± 0.03), 2: 1.27 (± 0.09), 3: 1.85 (± 0.19), 4: 2.62 (± 0.32), and 5: 3.66 (± 0.43). (**D**) Exploration errors, distractor errors, and rule-breaking errors decreased over trials. Data were fit with an exponential decay function ($y= a*\exp\left( -bx \right)+c$). (**E**) Mean (and 95% CI) of the decay factor (b) for each error type: Exploration error (0.14 [0.12, 0.17]), Rule breaking error (0.0041 [0.0025, 0.0058]), Distractor error (0.15 [0.12, 0.19]), and Perseverative error (0.022 [0.014, 0.029]). Exploration and distractor errors had a similar decay rate (p = 0.66). Stars denote significance levels for pairwise comparisons (Welch's t-tests). Decay factors for all error types were significantly greater than zero, confirming a decrease in all error types over the course of learning (t-test against zero, p-values: Exploration error: 6.6 × 10^-18; Rule breaking error: 5.4 × 10^-6; Distractor error: 7.3 × 10^-16; Perseverative error: 4.3 × 10^-8).
