# Supplementary material for: Non-human primates can flexibly learn serial sequences and reorder context-dependent object sequences
Source: PLoS Biol. 2025 Jun 23;23(6):e3003255. doi: 10.1371/journal.pbio.3003255 (PMC12208462; doi:10.1371/journal.pbio.3003255)
Supplement: S2 Fig — (A) Subjects reached 80% completion rate earlier in context 2 (yellow). (B) Trials to reach 80% completion (learning speed) in context 1 and 2. (C) Average reaction times for correct touches before and after learning point. Before learning points, ordinal position 1–5, respectively (Mean ± 95%CI): 0.84 ± 0.06, 1.14 ± 0.06, 0.57 ± 0.04, 0.59 ± 0.04, 0.62 ± 0.04; After learning points: 0.66 ± 0.04, 0.76 ± 0.05, 0.72 ± 0.05, 0.94 ± 0.06, 0.86 ± 0.05. Welch’s t test was applied to each ordinal position, and significant difference was found at the first and the last ordinal positions (Position 1: p < 0.00001, Position 2: p = 0.5520, Position 3: p = 0.2550, Position 4: p = 0.3416, Position 5: p = 0.0384). (D) Average reaction times for correct touches before (blue) and after swap (yellow) for each subject. No significant difference was found in all ordinal positions. The data underlying this figure can be found in the S1 Data file. (DOCX) [file pbio.3003255.s002.docx]

**Reaction time results**

**
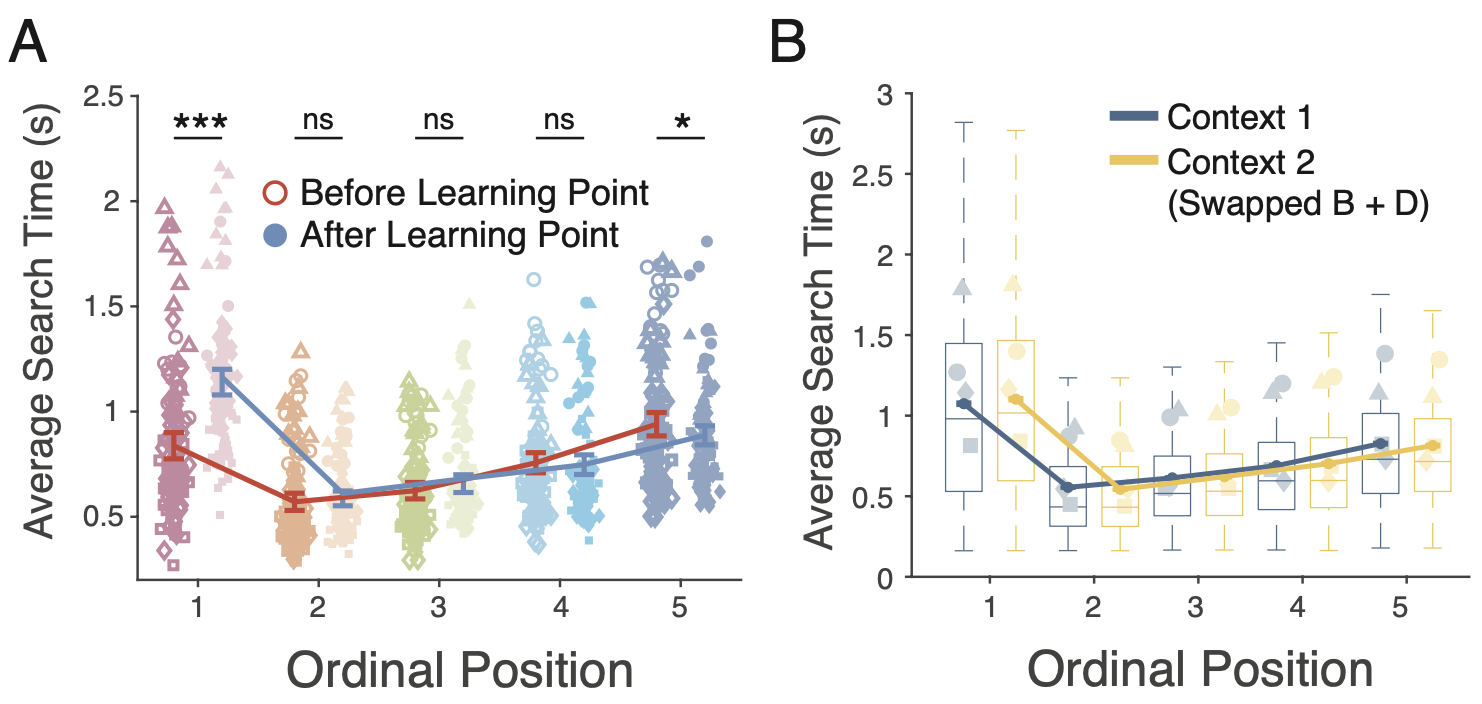
**

**S2 Fig.** **Reaction times to objects across ordinal positions during and after sequence learning.** (**A**) Average reaction times for correct touches before and after learning point. Before learning points, ordinal position 1 to 5 respectively (Mean ± 95%CI): 0.84 ± 0.06, 1.14 ± 0.06, 0.57 ± 0.04, 0.59 ± 0.04, 0.62 ± 0.04; After learning points: 0.66 ± 0.04, 0.76 ± 0.05, 0.72 ± 0.05, 0.94 ± 0.06, 0.86 ± 0.05. Welch’s t-test was applied to each ordinal position, and significant difference was found at the first and the last ordinal positions (Position 1: *p* < 0.00001, Position 2: *p =* 0.5520, Position 3: *p =* 0.2550, Position 4: *p =* 0.3416, Position 5: *p =* 0.0384). (**B**) Average reaction times for correct touches before (blue) and after swap (yellow) for each subject. No significant difference was found in all ordinal positions.
