# Supplementary material for: Non-human primates can flexibly learn serial sequences and reorder context-dependent object sequences
Source: PLoS Biol. 2025 Jun 23;23(6):e3003255. doi: 10.1371/journal.pbio.3003255 (PMC12208462; doi:10.1371/journal.pbio.3003255)
Supplement: S3 Fig — (A) Sequences (here: context 1 sequences only) repeated after the working memory task are performed better. In the new early condition, the 80% completion rate was reached after 3.93 ± 0.50 trials (Mean ± 95%CI), whereas, in the repeat condition, it was significantly quicker at just 2.13 ± 0.32 trials (New early versus Repeat: p: 1.19 × 10^−8^). For new context 1 sequences introduced late (to control for ‘time in the session’), the avg. trial to reach the 80% criterion was 2.87 ± 0.38, demonstrating that while there was some improvement in performance for new sequences in the later set, the most substantial gain was observed for the repeated sequences (New early versus New late: p: 0.0014; Repeat versus New late: p: 0.0052). (B) Choice reaction times for correct choices at each ordinal position were calculated to check if there were any difference in three conditions (New early: 0.79 ± 0.04; Repeat: 0.81 ± 0.04; New late: 0.77 ± 0.031). We found a slightly slower reaction time for the repeat blocks when compared with New late blocks (p: 0.039), but no other comparisons were significant (New early versus Repeat: p: 0.33; New early versus New late: p: 0.35). The data underlying this figure can be found in the S1 Data file. (DOCX) [file pbio.3003255.s003.docx]

**Effect of memory on sequence learning**

**
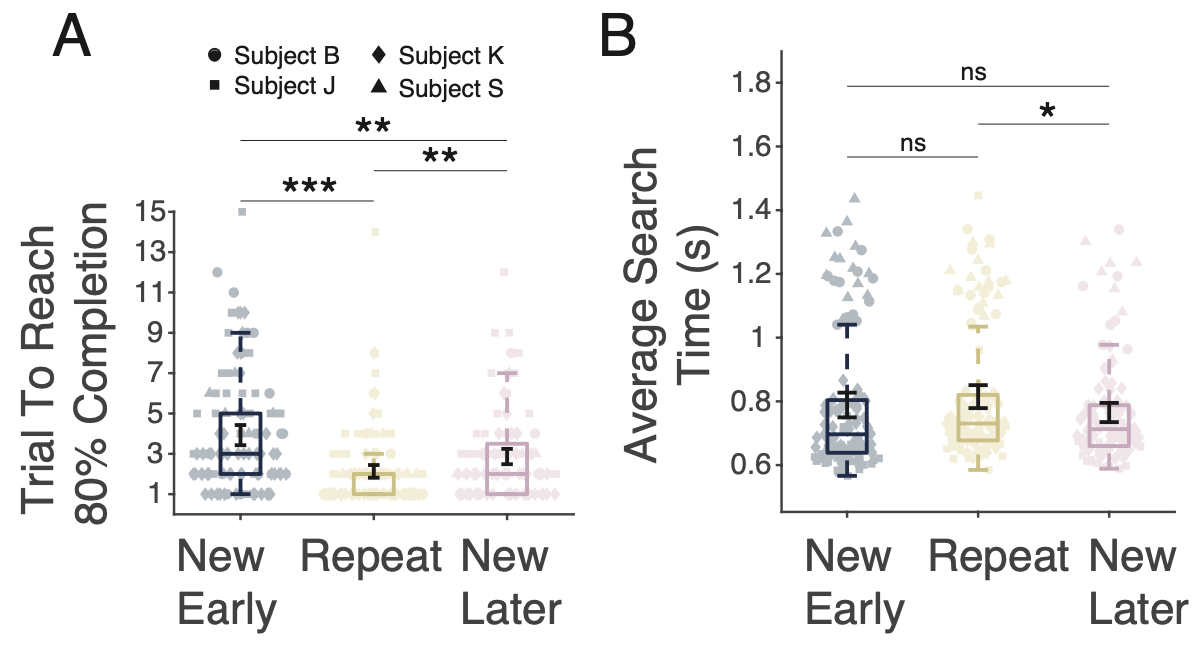
**

**S3 Fig.** **Memory of sequences improved performance.** (**A**) Sequences repeated after the working memory task are performed better. In the new early condition, the 80% completion rate was reached after 3.93 ± 0.50 trials (Mean ± 95%CI), whereas, in the repeat condition, it was significantly quicker at just 2.13 ± 0.32 trials (New early vs Repeat: p: 1.19 × 10^-8). For new sequences introduced late (to control for ‘time in the session’), the avg. trial to reach the 80% criterion was 2.87 ± 0.38, demonstrating that while there was some improvement in performance for new sequences in the later set, the most substantial gain was observed for the repeated sequences (New early vs New late: p: 0.0014; Repeat vs New late: p: 0.0052). (**B**) Choice reaction times for correct choices at each ordinal position were calculated to check if there were any difference in three conditions (New early: 0.79 ± 0.04; Repeat: 0.81 ± 0.04; New late: 0.77 ± 0.031). We found a slightly slower reaction time for the repeat blocks when compared with New late blocks (p: 0.039), but no other comparisons were significant (New early vs Repeat: p: 0.33; New early vs New late: p: 0.35).
