# Supplementary material for: Non-human primates can flexibly learn serial sequences and reorder context-dependent object sequences
Source: PLoS Biol. 2025 Jun 23;23(6):e3003255. doi: 10.1371/journal.pbio.3003255 (PMC12208462; doi:10.1371/journal.pbio.3003255)
Supplement: S4 Fig — (A) Task performance across varying object feature similarities and delay times. Objects shared features in either two feature dimensions (high similarity), or did not share features of any dimension (low similarity). Chance performance was set at 0.33. Accuracy was significantly higher for the Low Similarity condition compared to the High Similarity condition. High Similarity condition: Intercept: 0.4606; Slope: 0.0097; p = 0.6670; Low Similarity condition: Intercept: 0.7172; Slope: −0.0175; p = 0.3772. The overall average accuracy across all conditions was 0.59 ± 0.16 (Mean ± 95% CI), notably above chance (0.33). For the 0.50 s delay condition, performance was 0.47 ± 0.12 (High Similarity) and 0.71 ± 0.22 (Low Similarity); for the 1.25 s delay condition, it was 0.46 ± 0.12 (High Similarity) and 0.70 ± 0.19 (Low Similarity); for the 1.75 s delay condition, 0.48 ± 0.14 (High Similarity) and 0.68 ± 0.18 (Low Similarity). (B) Individual subject performance across all similarity and delay time conditions. Subject-level data remained consistent, with similar patterns observed between high and low similarity conditions, regardless of delay time. The data underlying this figure can be found in the S1 Data file. (DOCX) [file pbio.3003255.s004.docx]

**Working memory performance**

**
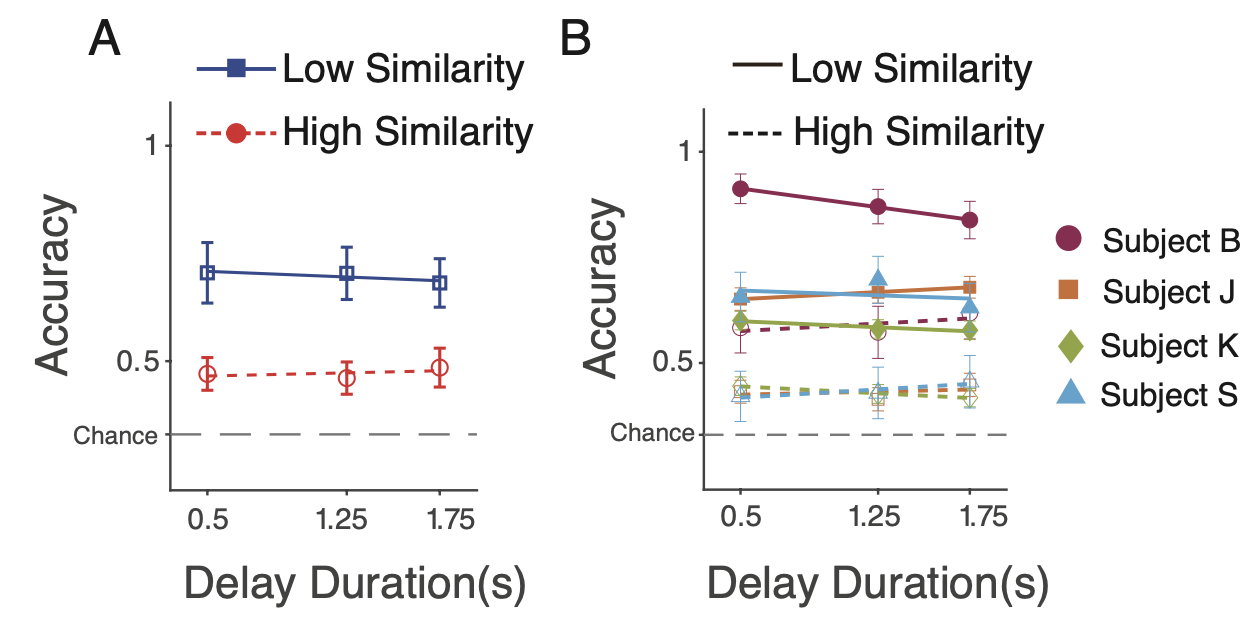
**

**S4 Fig.** **Working memory (delayed match-to-sample) performance.** (**A**) Task performance across varying object feature similarities and delay times. Chance performance was set at 0.33. Accuracy was significantly higher for the Low Similarity condition compared to the High Similarity condition. High Similarity condition: Intercept: 0.4606; Slope: 0.0097; p = 0.6670; Low Similarity condition: Intercept: 0.7172; Slope: -0.0175; p = 0.3772. The overall average accuracy across all conditions was 0.59 ± 0.16 (Mean ± 95% CI), notably above chance (0.33). For the 0.50s delay condition, performance was 0.47 ± 0.12 (High Similarity) and 0.71 ± 0.22 (Low Similarity); for the 1.25s delay condition, it was 0.46 ± 0.12 (High Similarity) and 0.70 ± 0.19 (Low Similarity); for the 1.75s delay condition, 0.48 ± 0.14 (High Similarity) and 0.68 ± 0.18 (Low Similarity). (**B**) Individual subject performance across all similarity and delay time conditions. Subject-level data remained consistent, with similar patterns observed between high and low similarity conditions, regardless of delay time.
