# Supplementary material for: Non-human primates can flexibly learn serial sequences and reorder context-dependent object sequences
Source: PLoS Biol. 2025 Jun 23;23(6):e3003255. doi: 10.1371/journal.pbio.3003255 (PMC12208462; doi:10.1371/journal.pbio.3003255)
Supplement: S5 Fig — (A) Proportion of distractor choices across trials in context 1 (left panel) and context 2 (right panel). The mean value is shown as the rightmost data point (Mean ± 95% CI, Before swap: 0.090 ± 0.010; After swap: 0.061 ± 0.006). (B) Same format as A for distractor choices in sequences early (left) and late (right) in the session. The average is shown as rightmost data point (Mean ± 95% CI, Initial encounter: 0.076 ± 0.008; Repetition: 0.046 ± 0.004). (C) Distractor choices at each ordinal position in context 1 (distractor is similar to B at the second ordinal position) and in context 2 (distractor is similar to B at the fourth ordinal position). Two-proportion Z-tests were applied at each ordinal position for comparing the difference. Stars denote sign. level. (Ordinal Position: Before and After Swap, Mean ± 95% CI) 1: 0.13 ± 0.01; 1: 0.08 ± 0.01; 2: 0.29 ± 0.01; 2: 0.20 ± 0.01; 3: 0.19 ± 0.01; 3: 0.22 ± 0.01; 4: 0.22 ± 0.01; 4: 0.34 ± 0.01; 5: 0.17 ± 0.01; 5: 0.16 ± 0.01. The data underlying this figure can be found in the S1 Data file. (DOCX) [file pbio.3003255.s005.docx]

**Learning to ignore distractor**

**
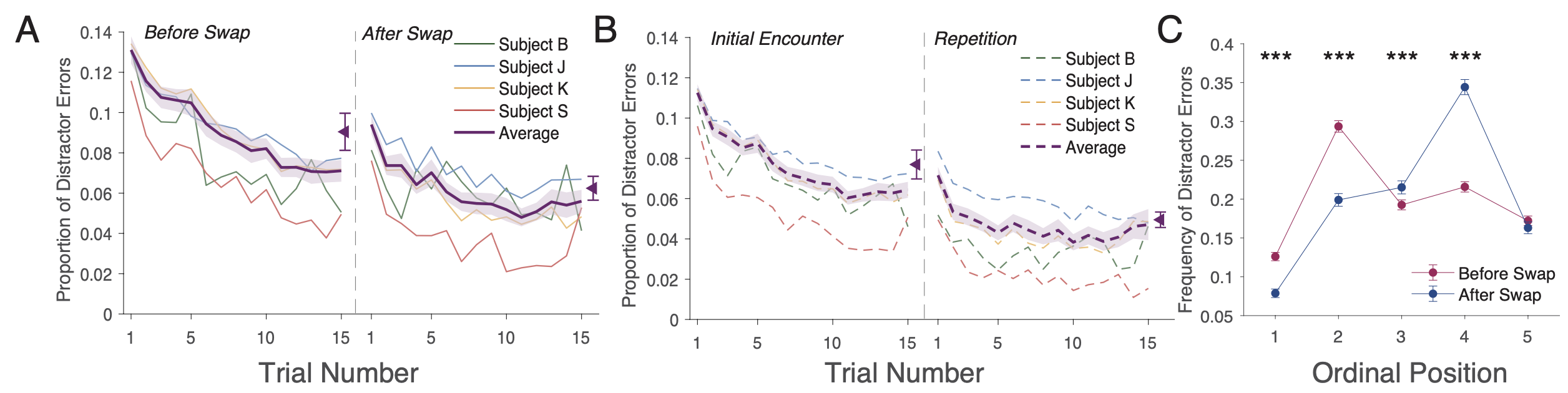
 S5 Fig.** **Learning to ignore distractor object.** (**A**) Proportion of distractor choices across trials in context 1 (*left* panel) and context 2 (*right* panel). The mean value is shown as the rightmost data point (Mean ± 95% CI, Before swap: 0.090 ± 0.010; After swap: 0.061 ± 0.006). (**B**) Same format as A for distractor choices in sequences early (left) and late (right) in the session. The average is shown as rightmost data point (Mean ± 95% CI, Initial encounter: 0.076 ± 0.008; Repetition: 0.046 ± 0.004). (**C**) Distractor choices at each ordinal position in context 1 (distractor is similar to B at the 2^nd^ ordinal position) and in context 2 (distractor is similar to B at the 4^th^ ordinal position). Two-proportion Z-tests were applied at each ordinal position for comparing the difference. Stars denote sign. level. (Ordinal Position: Before & After Swap, Mean ± 95%CI) 1: 0.13 ± 0.01; 1: 0.08 ± 0.01; 2: 0.29 ± 0.01; 2: 0.20 ± 0.01; 3: 0.19 ± 0.01; 3: 0.22 ± 0.01; 4: 0.22 ± 0.01; 4: 0.34 ± 0.01; 5: 0.17 ± 0.01; 5: 0.16 ± 0.01.
