# Supplementary material for: Non-human primates can flexibly learn serial sequences and reorder context-dependent object sequences
Source: PLoS Biol. 2025 Jun 23;23(6):e3003255. doi: 10.1371/journal.pbio.3003255 (PMC12208462; doi:10.1371/journal.pbio.3003255)
Supplement: S6 Fig — (A) Running means of posterior theta over iterations for each subject and each object. All values stable after burn-in. (B) Gelman–Rubin diagnostics for each subject and each object. All values are less than 1.1 representing good convergence. The data underlying this figure can be found in the S1 Data file. (DOCX) [file pbio.3003255.s006.docx]

**Bayesian statistics: convergence diagnostics**

**
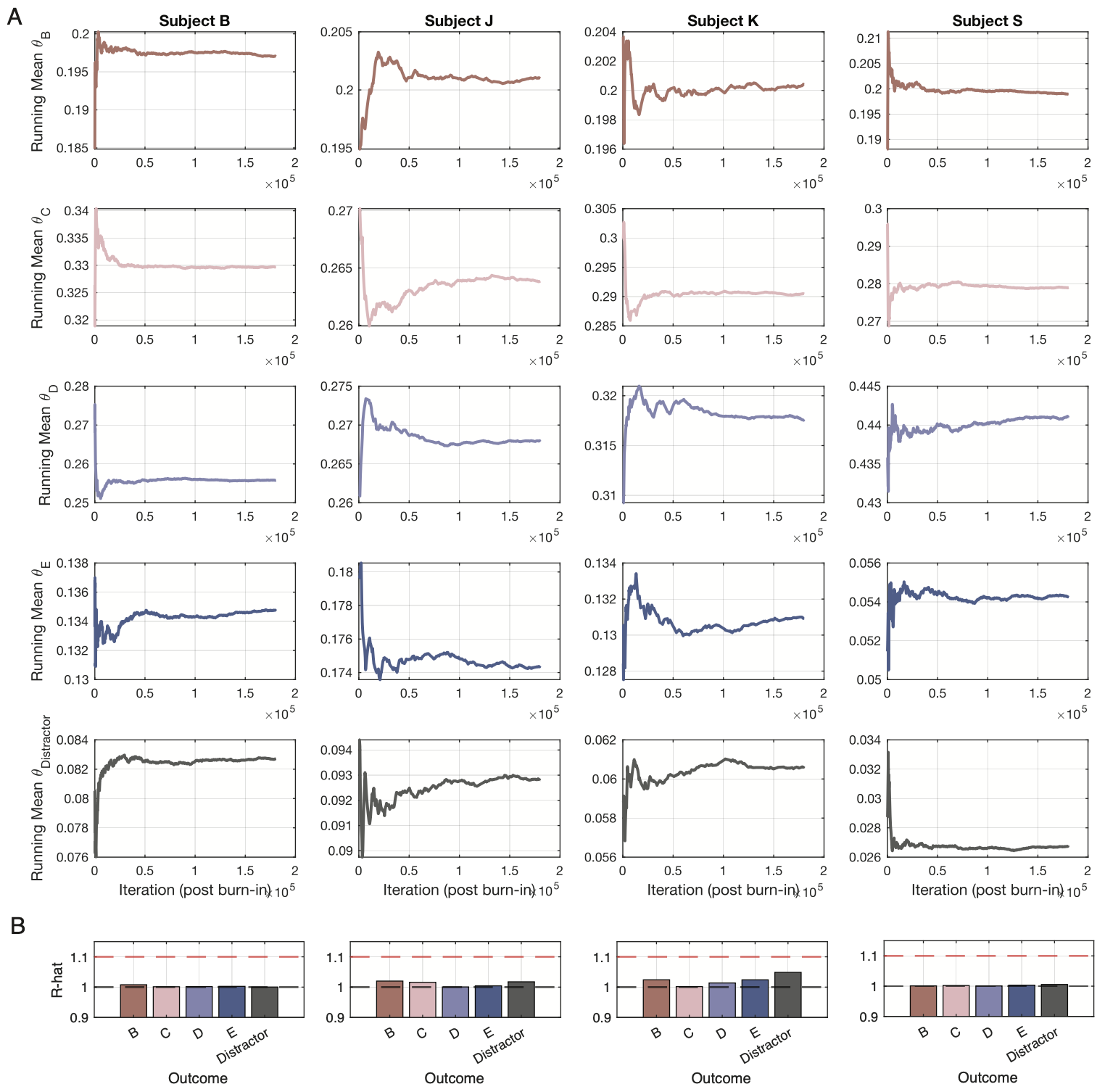
 S6 Fig.** **Convergence diagnostics of Metropolis–Hastings Markov chain Monte Carlo (MCMC) sampling**. (**A**) Running means of posterior theta over iterations for each subject and each object. All values stable after burn-in. (**B**) Gelmen-Rubin diagnostics for each subject and each object. All values are less than 1.1 representing good convergence.

**S1 Movie. Example performance of the context-dependent object sequence learning task.** Nonhuman primates (rhesus macaques) performing trials of the 5-object sequence learning task and adjusting to the swapped object order when the context changes from context 1 to context 2. The left side of the display illustrates the correct sequential order (left top) and the display visible to the experimenter that mirrors the main events of the screen (left bottom). The visual displays and task is controlled by the M-USE software platform. The right display shows a back view from inside the animal cage while the subject engages with a touchscreen station. The touchscreen station is mounted to one apartment cage compartment. The subject has free access mounted touchscreen station for ~90-120 minutes each week-day to engage with the task. Subjects hold their hand and mouth close to a stainless-steel sipper tube that delivers fluid rewards once a 5-object sequence is completed. The slider position of the progress bar on top of the screen steps forward with each correct choice and resets after errors.

The movie has two parts. The first part shows example performance of subject ‘NHP-K’ of a sequence after the subject reached the learning criterion (completing sequences in >80% of trials). Then, performance is shown for a swapped sequence in context 2 (a blueish context 1 background switches to a green context 2 background). The second part of the movie (starting at ~1:08 min) shows the example performance of subject ‘NHP-J’ for a context 1 and then a context 2 sequence.
