# Supplementary material for: Non-human primates can flexibly learn serial sequences and reorder context-dependent object sequences
Source: PLoS Biol. 2025 Jun 23;23(6):e3003255. doi: 10.1371/journal.pbio.3003255 (PMC12208462; doi:10.1371/journal.pbio.3003255)
Supplement: Attachment — Submitted filename: Reply_to_Reviewers_05.docx [file pbio.3003255.s011.docx]

Dear Reviewers and Editor,

We are grateful for the constructive reviewer comments that we address with additional analysis results, additional discussion, new figure panels and clarifications.

The revisions improved and expanded the paper which now represents a critical conceptual advance by showing that rhesus monkeys are able to anticipate the need to re-order the order of objects. This finding documents a high degree of cognitive flexibility which suggests that the nonhuman primates infer the temporal order of object sequences as opposed to be limited to learn chains of associations between neighboring items.

The revisions did not alter the original findings but increased the clarity, comprehensiveness and rigor in arriving at the main conclusions.

We summarize the main changes in brief bullet point format here (and all are in red font in the revised manuscript):

• We added Bayesian analysis for individual subjects’ performance to quantify that the swapping performance (re-ordering of non-adjacent objects) is the prevalent strategy for three of the four nonhuman primates. The new **Figure 3A-H** address the reviewer request to justify using n=4 subjects, which is ok for studies interested in individual within-subject studies (as compared to studies interested in the group-level).

• We quantified the choice strategies in individual sessions to evaluate how prevalent the swapping (position coding) strategy was. New **Figure 3I** visualizes that swapping was the dominant strategy in ~66% of experimental sessions.

• We adjusted the text to make it more readable for a general audience and more concise with regard to the design and statistical analysis. We also added conceptual clarifications in the discussion, especially by clarifying how working memory processes may underly successful mental re-indexing behavior.

• All data are now provided at an Open Science Framework repository: (https://osf.io/2vk97/ , DOI: 10.17605/OSF.IO/2VK97) and analysis code for reproducing results shown in each figures is now available on GitHub (<https://github.com/xwen1765/Re-ordering-of-Sequential-Order-in-Nonhuman-Primates>).

• We clarify that **Movie S1** is part of the submission, recognizing that it may not have been available to the reviewers.

Please see our **point-by-point replies** to the numbered reviewer comments below in *blue font*.

Thank you on behalf of all co-authors,

Thilo Womelsdorf &
Xuan Wen

**Reviewer #1:**

__________________________________________________________________________
**Comment #1:** These experiments show that macaque monkeys are capable of learning abstract slots or positions in a sequence.  The evidence is ability to obey a general rule "swap slot 2 with slot 4".  On the whole, I find the conclusion to be well supported, and some aspects of the results (e.g. specific link between working memory and rule following) are quite intriguing.  On the whole, though, I feel that abstract serial position slots in animals are already well supported by physiological data for the monkey (papers from Wang group cited here) and mouse (e.g. Behrens group).  I am not sure that enough is added by these behavioral data to justify publication in this journal.

*Reply:* We thank the reviewer for the positive and constructive comments. We adjusted the text to describe more explicitly the uniqueness of our findings. The assumption of the reviewer that abstract position coding has been shown in animals is based on neurophysiological evidence. The behavioral evidence has been either absent or it has been rather weak:

1) In the mouse, there is no evidence that objects are coded in serial order and can be flexibly swapped. The Behrens group is describing cognitive map structures in mice based on spatial navigation tasks and describe the implications of grid-, place-, and head- direction cells for those spatial cognitive map structures, but without behavioral evidence this information is used for manipulating object order flexibly
2) In the nonhuman primate, evidence that objects are coded in abstract positions is rejected by the dominant serial chaining model (summarized in the introduction). The Wang group has a special status by demonstrating that neuronal population responses are consistent with swapping object positions, but this neurophysiological evidence is limited to ordering the location of 2 items. Behaviorally they show that nonhuman primates can recall backwards 2- and 3-item sequences, and it is not described which factors underly the successful behavior, which could well entail primacy and recency effects which are memory biases that are independent of learning sequential structures. We are great admirers of Dr. Wang’s work and use the Wang findings as a major framework to understand the swapping of non-adjacent 5-objects and emphasize it more explicitly in the revised manuscript. We write e.g. …

*“While these neuronal findings are limited to playing backwards adjacent items, we suggest that they provide a versatile framework to conceptualize the re-indexing of objects to non-adjacent positions in our study.”* (Discussion section, line 442)

We adjusted the revised text to make explicit the uniqueness of our findings and how they relate to the Wang findings and the cognitive map (Behrens) literature. E.g. we now write in the discussion:

*“Our results are consistent with this framework and suggest that NHPs are able to utilize these neuronal re-indexing processes when learning to swap objects to non-adjacent sequential positions. This suggestion goes beyond frameworks, such as the serial chaining framework, that suggest that sequences and sequential re-ordering involves the formation of new serial chains of item-item associations. Taken together, the prevalence of pro-active swapping of non-adjacent object positions in our study documents a high level of flexibility of mental operations consistent with a re-indexing of objects to an abstract sequential structure. In our study, the sequential structure is a straight line of item positions to which objects are indexed. Such a line constitutes one of the simplest forms of a non-spatial cognitive map, implicated to be the cornerstone of flexible cognition (Rueckemann et al., 2021; Whittington et al., 2022; Whittington et al., 2025).”* (Discussion section, line 446)

__________________________________________________________________________
**Comment #2:** I also have two relatively major (though addressable) concerns.  First, I don't think a comparison of learning rates in contexts 1 and 2 (e. g. line 169 onward) is very telling - animals have already learned several things in context 1, so it is really not surprising that there is some generalization to context 2.

*Reply:* We agree and apologize if this analysis was presented not sufficiently clear (we had no intention to convey this as a main result). In the revised paper we clarify more explicitly that the good performance in context 2 is expected. We feel compelled to describe these results as a control analysis to document that neither monkey erroneously treated context 2 as containing an entirely new sequence. We moved the result figures from the main figure 2 into the supplementary material and summarize the results by describing

*“Overall, subjects initially dropped in performance in context 2 for a few trials but then adjusted to the swapped sequence in context 2 quickly, completing the A-D-C-B-E sequences in context 2 at 80% completion rate on average at trial 3.43 (± 0.56, Mean ± 95% CI), compared to 6.62 (± 0.72) trials for the initial learning of the A-B-C-D-E sequences in context 1 (Suppl. Fig. S2A*).” (Results, line 204)

__________________________________________________________________________
**Comment #3:**Similarly, I don't think it is very useful to show that correct swapping is more common when a sequence was previously learned, earlier in the session (section beginning line 254) - that earlier experience was for both contexts 1 and 2, so of course, context 2 (with swapping) also gains from that earlier experience.

*Reply:* Here, the reviewer refers to the finding that subjects showed better pro-active swapping performance for context 2 sequences that were repeated in the second half of the experiment.

The comment of the reviewer suggests that the finding is trivial that pro-active swapping is improved for repeated sequences, because the sequence was previously learned, and pro-active swapping was already evident when the sequence was shown for the first time in the first half of the session.

We think there could have been several different outcomes that would have been possible: Monkeys may …

(i) not have shown any long-term memory in the repeat sequence and showed the same pro-active and retro-active swapping (which would have indicated that the sequence choices were not memorized in longer term memory structures); or
(ii) they could have shown dramatically better pro- and retro-active swapping behavior in the repeated sequences (which we had hoped to see), or they could have
(iii) shown better pro-active swapping but no difference of retro-active swapping (which would have suggested that memory helped them to anticipate better the swapping requirement), or they could show
(iv) improved retro-active swapping while pro-active swapping did not change.

The results were consistent with possibility (iv): there was only a statistical trend to improve the anticipation of swapped positions (proactive swapping) but significant improvement to swap once an error was committed at the 2^nd^ ordinal position (retro-active swapping).

We adjusted the text to describe the design and the pattern of results more concisely and explicit. We write …

*“Subjects performed five to six context 1+2 sequence pairs in this second half of the experimental sessions (****Fig. 1B****). Half of these ‘late’ context 1+2 sequence pairs were novel (‘New Late’ sequences), but the other half were identical to a context 1+2 sequence pair that was shown prior to the delayed match-to-sample task (‘Repeat’ sequences). If long-term memory facilitated performance and swapping of positions B and D in context 2 of the late sequences, then we expect that late, repeated sequences were performed better and showed more prominent pro-active swapping than late, new sequences. We found that subjects reached the 80% completion rate already after on average 1.29 (± 0.13 95% CI) trials for repeated sequences, which was faster than the on average 3.5 (± 0.54) trials subjects needed to learn the same sequence early in the session, and it was also faster than the 2.90 (± 0.47) trials needed to reach 8-% completion rate for new sequences that were interspersed late in the session (‘New Late’ sequences) to control for a possible effect of the time-in-task (****Fig. 5A****;* ***Suppl. Fig. S3****). The likelihood of pro-active swapping was already above chance for context 2 of the early sequences and did only marginally increase further in repeated sequences, evident as a statistical trend (New Early vs. Repeat: p = 0.0717; New Early vs. New Late: p = 0.1178 Repeat vs. New Late: p = 0.7913;* ***Fig. 5B****). However, in repeated sequences subjects were significantly more likely in context 2 to retro-actively swap object D into the second position after erroneously choosing object B at the 2^nd^ ordinal position (New Early vs. Repeat: p = 0.0014; New Early vs. New Late: p = 0.0020; Repeat vs. New Late: p = 0.3133;* ***Fig. 5C****). This pattern of results shows that memory of the sequence from earlier in the session facilitated error correction in trials when subjects failed to pro-actively swap object D into the second ordinal position.”* (Results section, lines 326)

We think this is an interesting finding because it suggests that cognitive mechanisms related to “error correction” and “feedback monitoring” are facilitates efficient swapping behavior. These processes go beyond assuming that structures supporting working memory or long-term memory are sufficient to enable flexible re-ordering behavior.

__________________________________________________________________________
**Comment #4:** Minor comments:

Line 119.  I think "reset" is not a good word to use - presumably the counter does not go back to the start, as the animal is supposed to repeat just one previous choice?

*Reply*: Thank you for pointing us to this. They only had to re-do the previous choice as the reviewer mentions. We adjusted the text and write …

*“… subjects received for each correctly chosen object immediate visual feedback (yellow halo; for incorrect choices: grey halo) and an increment (for incorrect choices: a decrement) of the slider position that signaled how many steps away subjects were from receiving fluid reward (****Fig. 1A****, see* ***Movie S1****)”* (Results section line 138)

and

*“… the task paradigm provided immediate performance feedback of every choice in the form of the halo feedback and the stepping forward or backward of the slider position of the progress bar (****Fig. 1A****).”* (Discussion section line 549)

We also adjusted the figure legends accordingly.

__________________________________________________________________________
**Comment #5:** Line 133.  This is not what is shown in Figure 1C.

*Reply:* This was a wrong figure reference on our end. We corrected it. Thank you.

__________________________________________________________________________
**Comment #6:** Lines 149, Figure 1, Figure S1.  A variety of apparently different 80% rules are used - it is confusing, and perhaps needs more careful explanation of each.

*Reply:* We re-wrote and clarified that the 80% completion rate was used as learning criterion consistently across all analysis. We now write, e.g.

*“We found that all subjects performed above chance at each ordinal position documenting successful learning (proportion correct at ordinal position 1 (OP 1): 0.67 (mean) ± 0.026 (95% conf.); OP 2: 0.41 ± 0.016; OP 3: 0.55 ± 0.020; OP 4: 0.59 ± 0.017; OP 5: 0.79 ± 0.020) (****Fig. 1C****;* ***Suppl. Fig. S1A****). To assess the learning of a sequence we evaluated not individual ordinal positions but evaluated how likely subjects completed a full sequence by calculating how many trials the subjects needed to complete the 5-object sequences on average in 80% of trials above chance levels, which was with ten or less erroneous choices. Subjects reached this 80% completion rate on average within 6.62 ± 0.37 trials (subject B: 11.40 ± 1.23; J: 8.39 ± 0.58; K: 4.74 ± 0.40; S: 6.11 ± 1.03) (****Fig. 1D****).”* (Result section, line 176)

__________________________________________________________________________
**Comment #7:** Figure 1, Line 262.  It should be explicit that these data refer to context 1.  The reference to Figure 1 in line 133 is confusing since Figure 1 presents context 1 results.

*Reply:* We corrected this. Thank you for pointing us to this.

__________________________________________________________________________
**Comment #8:** Line 589.  Closing parenthesis missing.

*Reply:* This is now corrected.

__________________________________________________________________________
**Comment #9:** Line 740.  Figure 1E does not seem to show mean trials to reach completion.

*Reply:* This is now clarified.

__________________________________________________________________________
**Comment #10:** Figure 4E.  What are colours and dashed black line?

*Reply:* Thank you for pointing us to missing information in the legend – We updated the legends for these figure panels, which are now **Figure 5E** and **5G.** The different colors represent individual subjects in our experiment. The colored lines matching each point color represent the individual regression lines calculated separately for each subject. This dashed black line represents the overall regression calculated across all subjects' data points. The regression line illustrates the general relationship between the initial block completion rate and proactive swapping across the entire dataset.

__________________________________________________________________________
**Comment #11:** Figure S1.  Distractor error not defined here.

*Reply:* We added a definition in the legend, i.e. that distractor errors correspond to *“choosing the distractor object that was shown as a sequence-irrelevant 6^th^ object in the display”.*

__________________________________________________________________________
**Comment #12:** Figure S3.  Should specify this is for context 1.

*Reply:* We now specify this explicitly (thank you for recognizing the missing information).

__________________________________________________________________________
**Comment #13:** Figure S4.  Similarity not defined here.

*Reply:* Thank you. We added the definition of similarity in the methods and in the Figure S3 legend. E.g. we write in the methods

*“DMTS used Quaddle 1.0 stimuli that were composed of features from four feature dimensions (nine different body shapes, ten different arm styles, nine different body patterns, eight different color). In each block of the experiment, either two feature dimensions shared the same feature (high similarity), or none of the dimensions shared any features (low similarity).”* (Methods, Line 661)

**Reviewer #2**

__________________________________________________________________________
**Comment #14:** This study examines whether rhesus macaques can flexibly reorder learned object sequences based on contextual cues. While primates can swap adjacent items in sequences, it remains unclear if they can reassign objects to non-adjacent positions. Using a touchscreen task, macaques learned to order five objects and adjusted their responses when a background change signaled a new sequence. They successfully re-indexed objects, particularly when the original sequence was well-learned, and performance correlated with working memory. This study presents an interesting and valuable contribution to our understanding of cognitive flexibility in nonhuman primates. The authors provide novel insights into how primates infer and manipulate sequential structures. The findings challenge traditional views on sequence learning, suggesting that monkeys can go beyond simple associative chaining to infer latent serial structures. These results have important implications for research on primate
cognition, comparative psychology, and the evolutionary roots of structured thought.

However, there are several issues that I believe, once dealt with, could improve the manuscript. One issue is the current organization of the paper, where the methods appear at the end due to journal formatting. As a result, the experimental design is difficult to fully grasp until late in the manuscript, making it challenging for the reader to understand the logic behind the study. If restructuring is not possible, the authors should provide a more explicit description earlier in the manuscript, clearly explaining how sessions, sequences, and trials are structured - as they do L472-489. A more transparent outline of the learning process would significantly improve readability.

*Reply:* We thank the reviewer for the constructive and positive comments on our study. We adjusted the text of the revised manuscript to address the helpful comments.

We added in the introduction a more explicit description of the trial structure of the task and also refer to the Movie S1 earlier in the hope that the Movie S1 will be hyperlink that will be accessible to readers (it documents the task paradigm and the swapping responses). We introduce more explicitly the trial structure by writing:

*“The paradigm required NHPs to choose objects in a predetermined sequential order as shown in* ***Movie S1*** *(****Fig. 1A****). Objects were shown simultaneously on the screen. Subjects learned to sequential order five objects over successive trials. In each trial they received immediate visual feedback (a halo around the chosen object) about whether they chose an object at the correct ordinal position. When the choice was incorrect, they had to re-touch the last correctly chosen objects. In each trial they received fluid reward when they completed the five-object sequence. When they did not complete the sequence within 15 trials (maximally 10 errors) they did not receive fluid reward. When a sequence was completed, or the maximum of fifteen choices was reached the subjects were presented with a new trial in which the same objects were arranged at new locations to prevent a spatial strategy.”* (Introduction, Line 129)

We also introduce early in the text a new terminology “context 1+2 pair” to make explicit how the same objects from the sequence in context 1 were then re-configured in the subsequent context 2. We newly write in the revised introduction:

*“… the onset of the ‘context 2’ background served as a cue for the subjects to re-order the object sequence they had learned in the preceding 15 trials. The task paradigm allowed assessing the sequence learning (in context 1) and the re-ordering of the learned sequence (in context 2). A ‘context 1+2’ pair used a the same set of multidimensional objects (Wen et al., 2024). Ten to twelve unique ‘context 1+2’ pairs were evaluated in an individual experimental session that lasted ~90 minutes. Half-way through a session, after four or five context 1+2 sequence pairs, the subjects performed for 120 trials a delayed match-to-sample task that assessed short-term memory (****Fig. 1B****). After the delayed match-to-sample task subjects performed an additional five to six context 1+2 sequence pairs.”* (Introduction, Line 145)

We carefully went through the text and enhanced the clarity of the terminology and descriptions. The folder with the revised submission should contain a file with all changes highlighted in red font.

__________________________________________________________________________
**Comment #15:** Additionally, the writing could be more fluid and accessible. While the study deals with complex cognitive concepts, the occasional use of jargon makes certain sections dense. The authors could simplify some descriptions, use more commas to clarify sentence structure, and ensure that key points are conveyed as clearly as possible. 
To give an example, L105-109 could be clarified by (i) structuring the sentence as follow: "In computational models -COMMA- the latent temporal structure of experienced environments can be inferred and represented as a non-spatial cognitive map of item locations -FULL STOP-  Such representation enables the flexible re-indexing of objects to different positions in this cognitive map (Behrens et al., 2018; Whittington et al., 2022)" and (ii) expliciting the terminology used. A more reader-friendly presentation would make the study's significance and findings more approachable to a broader audience, including researchers outside the field of comparative cognition.

*Reply:* We agree and improved the revised manuscript text accordingly. We agree with the reviewer that there were many rather complex sentences, and the terminology was occasionally quite cryptic. The revised manuscript should be considerably improved.

With regard to the example sentence, we removed the term ‘*latent temporal structure*’ altogether and broke down the sentence into several sentences. We write:

*“In computational models such a temporal sequential structure of experienced environments can be described as a non-spatial cognitive map of item locations (Behrens et al., 2018; Whittington et al., 2022). A non-spatial cognitive map can be inferred from experiences and stored in synaptic weights in long-term memory, or they can be retained in ongoing activity slots using working memory (Whittington et al., 2025). We hypothesized that rhesus monkeys will be able to infer such a non-spatial structure from experience and that this structure will be revealed when they proactively re-order non-adjacent object items B and D to a novel A-D-C-B-E sequence. “ (Introduction, Line 116)*

Instead of ‘latent’ temporal structure we clarify more specifically how the ‘sequential’ structure could be considered an instance of a non-spatial cognitive map (which is the term used in the literature that we hope will be influenced by our findings). We define what we mean in the discussion section:

*“Taken together, the prevalence of pro-active swapping of non-adjacent object positions in our study documents a high level of flexibility of mental operations, consistent with a re-indexing of objects to an abstract sequential structure. In our study, the sequential structure is a straight line of item positions to which objects are indexed. Such a line constitutes one of the simplest forms of a non-spatial cognitive map, which is implicated to be the cornerstone of flexible cognition (Rueckemann et al., 2021; Whittington et al., 2022; Whittington et al., 2025).”* (Discussion section, line 471)

__________________________________________________________________________
**Comment #16:** The discussion on the neural basis of the cognitive operations described in the study is somewhat unclear. While drawing connections to neural mechanisms is valuable, it is not entirely evident how the behavioral findings directly map onto specific neural processes. The authors should clarify this link or consider whether it is necessary for the core argument of the paper.

*Reply:* We are grateful for the comment and adjusted the text at several places to address it. We think it is valuable to describe the recent set of studies by the Wang group on neuronal subspaces that temporarily emerged when monkeys retrieved a 2-item sequence backwards. This finding provides a parsimonious framework for understanding our evidence for pro-active swapping behavior.

However, the findings from the Wang group are presented in the literature as documenting that nonhuman primates show ‘flexible re-indexing’. We believe that the neuronal results suggests that this is a possibility, but that the behavioral data can be explained without such a neuronal mechanism (e.g. via backwards recalling serial chains of item-item associations).

In response to the reviewer comment we describe the neuronal data and how they may link to the behavior in our study more concisely, hoping this enhanced the clarity. We now write in the discussion section:

*“A neuronal correlate for such cue-triggered reconfiguration process has recently been suggested in an experiment that required NHPs to recall backwards the spatial order of 2- and 3-item sequences (Tian et al., 2024). Groups of frontal cortex neurons represented forward spatial 2-item sequences A-B until a cue instructed the subjects to recall the sequence backwards. During the re-coding of the forward into a backwards sequence neuronal population activity in frontal cortex transiently encoded both, the forward A-B sequence and the swapped items A as a rank 2 object and B as a rank 1 object. This transient encoding of both, forward and backwards item order was then followed by neuronal populations representing only the backward sequence that needed to be recalled (Tian et al., 2024). These findings suggest that swapping the order of adjacent objects involves a short-term memory that contains in separate neuronal populations the old item order (forward sequence) and the new item order (the backward item order).”* (Discussion section: line 440)

We also describe more explicitly that the findings could be a useful framework for understanding our results by writing:

*“While these neuronal findings are limited to playing backwards adjacent items, we suggest that they provide a versatile framework to conceptualize the re-indexing of objects to non-adjacent positions in our study.”* (Discussion section: line 450)

We also added a summary sentence of the neuronal findings that make them relevant for our study. We write:

*“There are two key insights of this framework. First, it suggests that the swapping operation involves the re-indexing of objects to their absolute serial position within a sequence, and second, that swapping involves the temporary short-term memory of old and new object positions in sequence.”* (Discussion section: line 459)

__________________________________________________________________________
**Comment #17:** Another concern is the small sample size. The study reports data from only four rhesus macaques, which limits the generalizability of the findings. It would be useful for the authors to clarify whether additional subjects were tested and, if so, why they were excluded. If only four monkeys were tested from the start, acknowledging this limitation more explicitly and discussing its implications for interpretation would be beneficial.

*Reply:* We had four animals available for this study, which is not unusual in studies with rhesus monkeys in this field. There were no other animals tested or excluded.

We clarify in the revised text that our study is not a ‘group study’ but rather extensively tests over multiple experimental sessions each of the individuals to infer how individual subjects solve the task (over multiple sessions). The consistency of effects of the individuals is then compared in a second step to fairly discuss the prevalent choice strategies across individuals. Consistent with this goal, we present the key results in detail for each of the animals and show in the figures individual monkeys (in different colors) in addition to the overall (combined) means.

To actively address the reviewer comment we adjusted the text and added statistical analysis:

First, we added a Bayesian analysis that quantifies the probability that individual monkeys showed the swapping strategy as the main strategy. Because there was one monkey who did not pass this test we aggregated the data to infer what the dominant strategy was across monkeys combined. We added new Figure 3A-H and summarize these results by writing: …

“*To address whether the proactive swapping strategy is consistent across subjects, we applied a hierarchical Bayesian model to estimate, for each individual, the probability of selecting objects B, C, or D immediately after choosing A in context 2 (****Fig. 3A-D****). For each of the subjects, the probability of choosing D at the 2^nd^ ordinal position was consistently higher than that of choosing object B, while in three of four subjects object D was chosen more likely than object C consistent with swapping as the dominant strategy (****Fig. 3E-H****; Subject B: B = 0.20 ± 0.01 (Mean ± SE), C = 0.33 ± 0.01, D = 0.26 ± 0.01; Subject J: B = 0.20 ± 0.01, C = 0.26 ± 0.01, D = 0.27 ± 0.01; Subject K: B = 0.20 ± 0.01, C = 0.29 ± 0.01, D = 0.32 ± 0.01; Subject S: B = 0.20 ± 0.02, C = 0.28 ± 0.02, D = 0.44 ± 0.02). One of the subjects (subject B) chose object C more likely than object D at the 2^nd^ ordinal position suggesting a predominance of the serial inference strategy over the swapping strategy. To evaluate whether the overall result pattern across subjects would distinguish the two choice strategies, we aggregated the results in context 2 across subjects and found that the posterior probability that the choice probability for D exceeded that for B was 0.99, and the posterior probability that choosing object D exceeded choosing C was 0.657. These results show the highest probability was that subjects choose D over both, objects B and C at the 2^nd^ ordinal position, consistent with pro-active swapping of object D into the 2^nd^ ordinal position being the prevalent strategy.”* (results, line 268)

To corroborate the finding that three of the four subjects applied the swapping strategy more often than the serial inference strategy we also evaluated how these strategies were deployed across individual experimental sessions. We summarize these new results by writing:

*“We further examined whether proactive swapping was the dominant retrieval strategy at the level of individual experimental sessions, or whether swapping was mixed with serial inference or other strategies. To address this, we fit three candidate strategies to the choice probabilities for objects B, C, and D in context 2 in each estimation:* *swapping positions, i.e. position coding (object D more likely being chosen than B and C), serial inference (object C more likely being chosen than B and D) and others (neither position coding nor serial inference) (****Fig. 3I****). The analysis revealed that swapping D and B as the choice strategy was favored in the majority of sessions, with 65.68% of sessions showing a posterior model probability for this strategy. The serial inference strategy accounted for 34.23% of sessions.”* (Results, line 286)

We acknowledge these findings explicitly in the discussion by writing that

*“…[w]hen a change in context indicated that non-adjacent objects B and D switched positions, three of four subjects swapped these objects more likely than erroneously choosing the next-ranked object C in the sequence (****Fig. 2B-F; 3****).”* (Discussion, line 410)

and

*“Consistent with the importance of successful memorization of the sequence in context 1 for swapping behavior, the one subject (subject B) who showed in context 2 swapping behavior less likely than a serial inference (i.e. continued choosing object C more likely than D after choosing A) was the subject with the poorest learning performance in context 1 by reaching 80% completion rate on average on trial 11.40 compared to 4.74-8.39 trials in the other monkeys (****Fig. 1D****).”* (Discussion, line 440)

We updated the method section to describe the procedures and provide the code for implementing the analysis.

We thank the reviewer for prompting us to add these analyses which are unusual in our field (where often only two rhesus monkeys are tested, e.g. the Wang studies cited as key references in the text) but add considerably information.

__________________________________________________________________________
**Comment #18:** The statistical analyses also require more transparency. The authors primarily use Welch's t-tests, but this method is not ideal for such a small sample size, particularly for multiple pairwise comparisons. 
At times, the manuscript simply states that a "t-test" was conducted without specifying whether it was Welch's or Student's (e.g. L559, 562), leading to some ambiguity. There are also inconsistencies in the choice of statistical tests—while paired t-tests are used in some comparisons (L582), they are not applied consistently. A more detailed explanation of why specific tests were chosen, along with a justification, is necessary.

*Reply:* We understand these points and clarified the statistical tests and provide more explicit justifications for using them.

First, we added a justification for using Welch’s t-test and added to the methods section:

*“To account for potential unequal variances in choice probability, we employed Welch's t-test. We used Bonferroni correction for correcting p-values for multiple comparisons or we used Benjamini-Hochberg FDR correction when this is explicitly stated.”* (Methods, line 696)

The sample sizes exceed the required sample sizes for these tests.

Secondly, we checked the text to explicitly state that we use Welch's t-test and apply Bonferroni correction for multiple comparisons (and not Student’s test). We selected the Bonferroni correction as a conservative way to control for Type I errors. For example, the legend for Figure 2B and 2D has been updated as follows:

*“(B) Probability of choosing objects immediately after erroneously choosing object B in context 2 … Three stars denote p < 0.001 difference (Welch's t-test with Bonferroni correction)….”* (Fig 2 legend, line 981)

The statistical results that are interpreted have typically high statistical significance. For example, the results in Figure 2 have the following specific p-values:

Figure 2B

Corrected P-value for Ordinal Position 3 vs 4 comparison: 0.0000000186

Corrected P-value for Ordinal Position 3 vs 5 comparison: 0.0000000000

Corrected P-value for Ordinal Position 4 vs 5 comparison: 0.0000000000

Figure 2D:

Context 1

Corrected P-value for Ordinal Position 2 vs 3: 0.025053

Corrected P-value for Ordinal Position 2 vs 4: 1.000000

Corrected P-value for Ordinal Position 3 vs 4: 0.082249

Context 2

Corrected P-value for Ordinal Position 2 vs 3: 0.000000

Corrected P-value for Ordinal Position 2 vs 4: 0.000000

Corrected P-value for Ordinal Position 3 vs 4: 0.175527

Difference Context 2-1

Corrected P-value for Ordinal Position 3 vs 4: 0.000854

Corrected P-value for Ordinal Position 3 vs 5: 0.000000

Corrected P-value for Ordinal Position 4 vs 5: 0.006832

The other analyses where multiple comparison correction was needed was for Fig 2E, and Figure 4A-I.

For Fig 4A-I, Benjamini-Hochberg false discovery rate (FDR) corrections were applied. In our specific analysis, the Bonferroni correction resulted in one pairwise comparison (Fig. 4F) approaching significance (p = 0.024, adjusted p = 0.072). This suggests a potential effect that might be deemed non-significant solely due to the conservative nature of the Bonferroni correction. The FDR approach, on the other hand, controls the expected proportion of false positives among the rejected hypotheses. This offers a more balanced approach, increasing the power to detect true effects while still providing a reasonable level of control over Type I errors.

Adjusted p-values in figure 4:

A:

B-C p-value: 0.1395888735

B-D p-value: 0.0000000155

C-D p-value: 0.0000088447

B:

B-C p-value: 0.0227673218

B-D p-value: 0.0227673218

C-D p-value: 0.9164936829

C:

B-C p-value: 0.0087037624

B-D p-value: 0.0000000246

C-D p-value: 0.0026985936

D:

B-C p-value: 0.0030631502

B-D p-value: 0.0000000273

C-D p-value: 0.0030631502

E:

B-C p-value: 0.0248129265

B-D p-value: 0.0248129265

C-D p-value: 0.8928911534

F:

B-C p-value: 0.0003690491

B-D p-value: 0.0000000144

C-D p-value: 0.0242319591

G:

B-C p-value: 0.1918518615

B-D p-value: 0.2103411096

C-D p-value: 0.7176908728

H:

B-C p-value: 0.7100615515

B-D p-value: 0.2064815616

C-D p-value: 0.2064815616

I:

B-C p-value: 0.3817752231

B-D p-value: 0.7937244542

C-D p-value: 0.3817752231

__________________________________________________________________________
**Comment #19:** Additionally, the manuscript would benefit from a clearer description of the statistical tools used, including the R functions and packages. Providing the R script would further enhance transparency and reproducibility. Given the small sample, Bayesian analyses might be a more robust alternative and should be considered.

*Reply:* We provide with the revised manuscript the analysis code (via a github link) and the data (on the open science platform OSF). We also added descriptions to the used statistical software package (Matlab) in the text. We write:

*“All analyses were performed using MATLAB 2024a (The Mathworks). The data supporting this study are openly available at the Open Science Framework repository: https://osf.io/2vk97/ (DOI: 10.17605/OSF.IO/2VK97). All analysis code for reproducing the main and supplementary figures is publicly available on GitHub:* [*https://github.com/xwen1765/Re-ordering-of-Sequential-Order-in-Nonhuman-Primates*](https://github.com/xwen1765/Re-ordering-of-Sequential-Order-in-Nonhuman-Primates)*.”* (Data availability section, line 825)

We also added Bayesian analysis as an additional means to quantify the probabilities of swapping at the level of (1) individual subjects, (2) aggregated across subjects, and (3) for quantifying the dominant choice strategy on a session-by-session level. The results are shown in a new Figure 3 and described in more detail in our reply to the previous comment #17. We also added four references that we used for the new Bayesian analysis.

The provided code shows that the Welch’s t-test was implemented in matlab using the function: ttest2(allProbsCombined(:, pos1), allProbsCombined(:, pos2), 'Vartype', 'unequal').

_________________________________________________________________________
**Comment #20:** Overall, this study presents interesting findings and contributes to our understanding of cognitive flexibility in primates. Addressing these concerns would significantly strengthen the paper and improve its accessibility to a broader scientific audience.

*Reply:* We thank the reviewer for the constructive and helpful comments. We believe the additional analysis and statistical results enhanced the study and more it comprehensive.

__________________________________________________________________________
**Comment #21:** Review by Maël Leroux, University of Rennes
In an effort to promote transparency and kindness in academic discourse, I have chosen to systematically sign my reviews. While I recognize that this may not be suitable for everyone, particularly early-career researchers, I believe that fostering respectful and constructive feedback is essential for advancing science and maintaining integrity within our community.

*Reply:* We agree and very much appreciate the open and constructive review comments.

**Reviewer #3**

__________________________________________________________________________
**Comment #22:** This paper presents a detailed experiment to investigate whether nonhuman primates can learn to re-order non-adjacent elements in an order element. I am not an expert on core cognition, so I will not focus my review on the experimental design - although it sounds robust, thoroughly designed and relevant to me-, but rather on the manuscript itself. 

I think that given PlosBiology is not a specialized journal and has a large audience, the manuscript would benefit from more pedagogical writing. Indeed, at the moment, I think that it is written more for an audience of experts, which makes it hard to follow in some places. In particular, the relevance of the study to science is not clear. I would appreciate a few sentences at the beginning of the intro and at the end of the discussion to frame why this was important to study. This does not need to be long, but 1-2 sentences with clear examples could be of good help to evaluate the impact of the paper. I also struggled to follow every step of the study, since some information about the design is missing. Given that this is a dense paper with lots of different analyses and results, I think it would be good to "take the reader by the hand"  a bit more and better explicit what was done, and why.

I also have questions about the statistics - I am not asking the authors to redo them, but to justify their choice - why did you use mostly non-parametric tests (Z-test, t-tests) and not models, where you could have controlled for the identity of the subject or the sequence? You also did not mention how you processed the experimental data (e.g., which software did you use?) and how you conducted the statistical analyses (R? If so, what packages?).

*Reply:* We thank the reviewer for the thoughtful and constructive comments. We adjusted the text to enhance conciseness and clarity at multiple places (and removed or defined vague terminologies like “latent structure”). All changes are highlighted in red font.

In the introduction, we more carefully describe the main design features of the task, and also added a longer introductory paragraph that contextualize the study and why this study is important. We write

*“Mental flexibility refers to the ability to arrange thoughts or actions in novel ways. Flexible mental operations are required for many serially ordered behaviors including communication, counting, problem solving, and foraging (Davis and Pérusse, 1988; McNamee et al., 2021). These serial behaviors benefit from knowing the underlying sequential structure that guides the ordering of information. In language systems, communication can rely on the flexible arrangement of words into sentences based on syntactical rule structure; Counting and mathematical operations rely on knowing the regular numbering systems; Solving problems is facilitated by the ordering of subgoals reflecting the problem structure; and foraging becomes efficient if a spatial map structure of patch locations is retained to ease choosing the right foraging patch. For all these examples, mental operations and behavior become more flexible when the underlying structure for sequencing information is known. Consistent with this suggestion, the ability to infer sequential structures from experiences and to mentally re-arrange information within that structure underlies measures of intelligence and is particularly well developed in humans compared to nonhuman primates (NHP) (Dehaene, 2021; Passingham, 2021). However, it has remained debated, how capable NHPs are to infer abstract sequential structure and whether they can use that structure to flexibly guide mental operations (Dehaene et al., 2015; Whittington et al., 2022; Leroux et al., 2023; Passingham and Lau, 2023).”* (Introduction, line 73)

We also addressed the question about the statistics in the detailed replies to the specific comments (below). We adjusted the manuscript to more explicitly describe when we used Welch’s test or linear models, why we added a new Bayesian statistic, and that we did not use other t-tests.

We also provide all data and analysis code in a github repository (linked in the Data Availability section)

Overall, we are deeply grateful to the reviewer whose comments triggered significant clarifications and improvements to the original manuscript.

__________________________________________________________________________
**Comment #23:**Below you'll find more specific comments:
L73 "Flexible mental operations": Maybe keep the same terminology to avoid confusion in the reader

*Reply:* We followed this suggestion and streamlined the terminology throughout the manuscript.

__________________________________________________________________________
**Comment #24:** L74-75: Maybe give an example in humans? It is not clear how these are necessarily ordered behaviours, and how flexible mental operations are crucial

*Reply:* We added examples of the structures and rule systems that underly flexible communication, counting, problem solving, and foraging and write in the revised manuscript:

*“Flexible mental operations are required for many serially ordered behaviors including communication, counting, problem solving, and foraging [1, 2]. These serial behaviors benefit from knowing the underlying sequential structure that guides the ordering of information. In language systems, communication can rely on the flexible arrangement of words into sentences based on syntactical rule structure; Counting and mathematical operations rely on knowing the regular numbering systems; Solving problems is facilitated by the ordering of subgoals reflecting the problem structure; and foraging becomes efficient if a spatial map structure of patch locations is retained to ease choosing the right foraging patch. For all these examples, mental operations and behavior become more flexible when the underlying structure for sequencing information is known. Consistent with this suggestion, the ability to infer sequential structures from experiences and to mentally re-arrange information within that structure underlies measures of intelligence and is particularly well developed in humans compared to nonhuman primates (NHP) [3, 4]. However, it has remained debated, how capable NHPs are to infer abstract sequential structure and whether they can use that structure to flexibly guide mental operations [5-8].”* (Introduction, line 75)

__________________________________________________________________________
**Comment #25:** L77 "latent temporal structure": I would detail what this is exactly, especially since it is a crucial concept in your study (maybe give an example?)

*Reply:* Thank you for pointing us to the suboptimal terminology - We removed the term ‘latent’ altogether from the abstract and the main text and more specifically say “sequential structure”. In the discussion section we then make the link to the literature on “cognitive map” by defining the sequential structure. We write:

*“Taken together, the prevalence of pro-active swapping of non-adjacent object positions in our study documents a high level of flexibility of mental operations, consistent with a re-indexing of objects to an abstract sequential structure. In our study, the sequential structure is a straight line of item positions to which objects are indexed. Such a line constitutes one of the simplest forms of a non-spatial cognitive map, which is implicated to be the cornerstone of flexible cognition (Rueckemann et al., 2021; Whittington et al., 2022; Whittington et al., 2025).”* (Discussion, line 494)

__________________________________________________________________________
**Comment #26:** L86: ordering of items?

*Reply:* Adjusted to *“serial ordering of visual objects”.*

__________________________________________________________________________
**Comment #27:** L91: what is a "reverse play 3-item sequence"?

*Reply:* Thank you for recognizing that this is an odd phrase. We removed “reverse” and consistently refer to it as *“recalling a sequence backwards”*, or as *“recall[ing] a sequence A-B-C backwards as C-B-A”.*

__________________________________________________________________________
**Comment #28:** L92-94: It is not clear how the studies you mention point to these limitations
L96: You say "This re-indexing can be achieved by swapping the relative rank of adjacent items" but in your example, A and C are not adjacent, right?

*Reply:* A and C are non-adjacent but retrieving A-B-C backwards as C-B-A can be achieved by switching the rank of neighboring (adjacent) items without knowledge of the ordinal positions. Please also consider that the Wang paper performed neuronal analysis only on the 2-item sequences (i.e. only A-B) so the main conclusions from these papers apply to short sequences only. We rephrased this aspect and write:

*“… the ability to recall a sequence A-B-C backwards as C-B-A involves the re-ordering of items to new positions in the sequence (Tian et al., 2024), but this re-ordering can be achieved by swapping the relative rank of adjacent items in a sequence without the need to represent an abstract sequential structure or ordinal positions to which items are flexibly assigned (Kao et al., 2020).”* (Introduction, line 112)

__________________________________________________________________________
**Comment #29:** L113-115: This sentence is a bit too long and complex

*Reply:* We shortened and rephrased this to:

*“The paradigm required NHPs to choose five objects in a predetermined sequential order as shown in* ***Movie S1*** *(****Fig. 1A****). Objects were shown simultaneously on the screen. Subjects learned the sequential order of the five objects over successive trials. In each trial they received immediate visual feedback …”* (Introduction line 138)

__________________________________________________________________________
**Comment #30:** L124-126: I am not sure, at this stage, what a session is. Does it mean that each individual had to learn a new pair for each new work session?

*Reply:* We revised the text to define sessions earlier in the text and make explicit how many pairs monkeys learned per session. This is an important and quite unique strength of the paradigm. We write:

*“… the onset of the ‘context 2’ background served as a cue for the subjects to re-order the object sequence they had learned in the preceding 15 trials. The task paradigm allowed assessing the sequence learning (in context 1) and the re-ordering of the learned sequence (in context 2). A ‘context 1+2’ pair used a the same set of multidimensional objects (Wen et al., 2024). Ten to twelve unique ‘context 1+2’ pairs were evaluated in an individual experimental session that lasted ~90 minutes. Half-way through a session, after four or five context 1+2 sequence pairs, the subjects performed for 120 trials a delayed match-to-sample task that assessed short-term memory (****Fig. 1B****). After the delayed match-to-sample task subjects performed an additional five to six context 1+2 sequence pairs.”* (Introduction line 161)

__________________________________________________________________________
**Comment #31:** L131 "by erroneously choosing the object C that is adjacent to object B in context 1": I am not sure what this mean, can you be more specific?

*Reply:* After introducing that subjects learned in context 1 to choose objects in the order A-B-C-D-E we revised the text and now write more concisely:

*“When the background changed to context 2 to indicate that the same objects are re-ordered to A-D-C-B-E they overall followed two different strategies in parallel. In some trials in context 2 they chose at the second serial position in context 2 the object C instead of B, i.e. they recognized that object B needed to be re-ordered but erroneously chose the next-in-line object C instead of the correct object D. The erroneous choice of C at the second ordinal position suggests subjects erroneously inferred the next adjacent objects in the series, i.e. they followed a serial inference strategy. In other trials in context 2 subjects correctly anticipated the swapped positions of objects B and D and chose object D at the 2^nd^ ordinal position. This pro-active swapping was more likely than a serial inference strategy when the initial sequence was learned better, and it correlated with working memory performance on a session-by-session level.”* (Introduction, line 172)

__________________________________________________________________________
**Comment #32:** L134 "serial inference strategy": What is this?

*Reply:* Please see the previous reply. The revised text defines the *serial inference strategy* explicitly already in the introduction.

__________________________________________________________________________
**Comment #33:** L141: In line with the previous comment, I would appreciate a bit more details about what a session is. Does it mean that monkeys had to learn several pairs? Or just one pair per ind, across several sessions?

*Reply:* Monkeys learned every day of the week (in every experimental session) 10-12 different sequences (each with new objects). Please also see reply to comment #34. We clarify in the text that

*“Ten to twelve unique ‘context 1+2’ pairs were evaluated in an individual experimental session that lasted ~90 minutes. Half-way through a session, after four or five context 1+2 sequence pairs, the subjects performed for 120 trials a delayed match-to-sample task that assessed short-term memory (****Fig. 1B****). After the delayed match-to-sample task subjects performed an additional five to six context 1+2 sequence pairs.”* (Introduction line 165)*”*

We also analyzed how the swapping performance varied over multiple sessions and write:

*“We found that pro-active swapping was apparent already in the first experimental sessions and gradually increased over sessions (R^2^: 0.0717, p=0.0030;* ***Fig. 5H****), while the likelihood of retro-active swapping remained similar across sessions (R^2^: 0.0108, p=0.2559) (****Fig. 5I****).”* (Results, line 391)

In response to another reviewer comment we also analyzed the choice strategies of monkeys in individual sessions and summarize the results in the revised text by writing that we

*“… examined whether proactive swapping was the dominant retrieval strategy at the level of individual experimental sessions, or whether swapping was mixed with serial inference or other strategies. To address this, we fit three candidate strategies to the choice probabilities for objects B, C, and D in context 2 in each estimation: swapping positions, i.e. position coding (object D more likely being chosen than B and C), serial inference (object C more likely being chosen than B and D) and others (neither position coding nor serial inference) (****Fig. 3I****). The analysis revealed that swapping D and B as the choice strategy was favored in the majority of sessions,”* (Results, line 303)

We also refer to the **Movie S1** earlier in the introduction, hoping that the movie is available as a hyperlink to the reviewers (and readers) to help disambiguating the design and see an illustration of the performance of the monkeys.

__________________________________________________________________________
**Comment #34:** L148-149: I don't understand this criteria. You are looking, in a sequence, for the first trial for which they have at least 80% of good answers? Or do you take 80% of all the trials with less than 10 errors, but then what do you do with them? Please clarify. I also think that 10 errors out of 15 choices is a lot and does not spontaneously look like success... Why this threshold?

*Reply:* Monkeys received fluid reward when they completed a sequence. We therefore use the completion of a sequence as a criterion for performance success (rather than the percent of correct choices of objects at a specific ordinal position).

The revised text more explicitly describes the different performance measures that we used. First, we describe that performance at each ordinal position was above chance level by writing:

*“We found that all subjects performed above chance at each ordinal position documenting successful learning (proportion correct at ordinal position 1 (OP 1): 0.67 (mean) ± 0.026 (95% conf.); OP 2: 0.41 ± 0.016; OP 3: 0.55 ± 0.020; OP 4: 0.59 ± 0.017; OP 5: 0.79 ± 0.020) (****Fig. 1C****;* ***Suppl. Fig. S1A****).”* (Results, Line 195)

When subjects were above chance level at each of the ordinal position, they completed the sequence. We therefore used an 80% completion (across trials) as a test statistics quantifying how fast they reached above chance-level for the majority of sequences. Figure 1D shows that the completion rate increases from ~50% in the first trial to >85% on trial 15 for each of the monkeys. The 80% completion criterion is the means to capture the upwards trend (the learning) with some criteria threshold that applicable to all individuals. We kindly refer to the ***Movie S1*** which documents implicitly how well subjects performed when they successfully reached the learning criterion.

The revised text describes more explicitly that we …

*“… assess[ed] the learning of a sequence we evaluated not individual ordinal positions but evaluated how likely subjects completed a full sequence by calculating how many trials the subjects needed to complete the 5-object sequences on average in 80% of trials above chance levels, which was with ten or less erroneous choices. Subjects reached this 80% completion rate on average within 6.62 ± 0.37 trials (subject B: 11.40 ± 1.23; J: 8.39 ± 0.58; K: 4.74 ± 0.40; S: 6.11 ± 1.03) (****Fig. 1D****). Sequence learning performance is visually shown in* *the* ***Movie S1****.”* (Results, Line 197)

Please also consider that the analysis of good versus poorly performance sequences were analyzed separately, showing that monkeys showed quite some variability in performance. The swapping behavior was evident in context 2 when the sequences were learned at a higher performance level (with < 5.5 errors on average).

__________________________________________________________________________
**Comment #35:** L153 "80% completion rate": Is this the same as the criteria above? Here, it sounds as if you look at the first trial for which you have 80% of correct choices, but that contradicts the "10 or less error" criteria, as 10 errors / 15 choices is less than 80% success. Also, I don't understand the link with the "less than 5 errors per trial", this is not 80% success. Thank you to clarify these

*Reply:* Thank you for these questions. We expand the text to clarify this procedure and write:

*“There was variability of learning, which allowed rank ordering the sequences by the average number of errors per trial across trials for each sequence and comparing learning for fewest versus most errors. For the top third of sequences with the least errors per trial (≤5.33 errors per trial) the 80% completion rate was achieved after 2.68 (± 0.20) trials, while the bottom third of sequences with the highest error rates (>6.6 errors per trial) reached 80% completion only after 9.25 (± 0.51) trials (****Figure 1E****).”* (Results, line 204)

In each trial, a subject could make up to 10 errors, and each sequence was performed for 15 trials. For this analysis we calculated the average number of errors per trial across the 15 trials for each sequence. We then ranked all sequences by this average low to high and defined the top one-third (lowest error rates) as “well-performed blocks” and the bottom one-third (highest error rates) as “poorly performed blocks.”

The ‘completion rate’ quantifies learning progression across trials of a sequence.

The rationale to rank order sequences according to their average number of errors is that for sequences with fewer average errors per trial, subjects tend to complete the sequence earlier, indicating faster learning progression (which is confirmed as shown in ***Figure 1E****)*.

__________________________________________________________________________
**Comment #36:** L181-182: This part is not clear to me. It sounds as if some subjects spontaneously tried B and then saw it did not work so they tried C because it is the closer, vs some subjects immediately tried D. That's probably not what happened, and it is not clear why they would choose D over E, for example. Please clarify this sentence.

*Reply:* Yes, this is what happened when the context 1 sequence was poorly learned. Monkeys only more likely “chose D after correctly choosing A” when they had learned the sequence in context 1 to a higher degree – otherwise erroneously choosing B, or erroneously choosing C were common. We define this erroneous “serial inference strategy” more explicitly in the revised text and clarify the text to:

*“A serial chaining framework predicts subjects will erroneously choose object B at the second ordinal position and adjust to this error by choosing the next neighboring object C at the third ordinal position because its relative position is closest to the second position, i.e. they inferred the serially next ordinals’ position object. We refer to this behavior as reflecting a serial inference strategy. In contrast to such serial inference, subjects may also at the second ordinal position pro-actively choose object D.” (Results, Line 236)*

__________________________________________________________________________
**Comment #37:** L184-186: I would also like to know, after they understood that D came second, what they did with B. Did they immediately assume that it was replacing D?

*Reply:* Yes, this is correct. We present an important control analysis in Figure 2E that shows that after choosing “D” at the 2^nd^ ordinal position they correctly chose object C (and not object E which would have been expected if they continued following a D-E sequence from context 1. We write:

*“One prediction of the re-indexing strategy is that once object D was correctly chosen in context 2 subjects should next choose object C, instead of erroneously choosing object E which followed object D in context 1. Consistent with this hypothesis subjects chose C more likely than object E following choosing D, i.e. they left object E at the 5^th^ ordinal position (****Fig. 2F****) (Welch’s t-test, p < 1 × 10^-10; correctly choosing object C: 0.415 ± 0.010, incorrectly choosing object E: 0.232 ± 0.009 and object B: 0.263 ± 0.008).”* (Results, Line 281)

__________________________________________________________________________
**Comment #38:** L188-190: I don't understand how they assumed this immediately. Was there a previous training before? Why would they go for D directly, instead of C and E for example? If this is not an immediate answer, then clarify this by stating after how many trials they adapt each strategy

*Reply:* When the context 1 changed to context 2 the monkeys had extensively learned that the same objects they had serially ordered in context 1 underwent a re-ordering (swapping B and D). We adjusted the text early in the results section to clarify that the context change was the cue for swapping, and that this context rule was trained for 10-12 sequences every session (every weekday). (please also see reply to comment #34).

We started data collection when animals showed stable performance and analyzed explicitly how the pro-active swapping was already evident in the first sessions (but gradually increased), while the retro-active swapping (which means they needed error feedback to be reminded of the swapping) remained similar across all sessions.

__________________________________________________________________________
**Comment #39:** L205-212: Over how many trials? Are you looking at all the trials, just the first ones? This needs to be clarified

*Reply:* We first analyzed context 2 behavior in trials while subjects had not yet reached the 80% completion rate criterion in context 2 (this is described in the text). In a second analysis we restricted the analysis to the “first choices” in context 2 as the reviewer suggests. We describe this analysis and the results by writing:

*“The swapping results so far indicate that subjects adjusted their choices in context 2 consistent with re-indexing objects D and B to different ordinal positions, but this re-indexing might depend on the efficient use of the immediate visual feedback subjects received for correct and erroneous choices. For example, when subjects chose object D at the correct 2^nd^ ordinal position in context 2, they received positive feedback, which on average will have increased the likelihood of continuing choosing object D as opposed to erroneously choosing object C at the correct 2^nd^ ordinal position. To control for this possibility and test whether the re-indexing was evident without having received feedback we performed a third analysis restricted to only the first choice of the first trial in context 2 at the 2^nd^ position (i.e. after object A was correctly chosen) and compared it with the first choice of the last trial in context 1 under the same condition (****Fig. 4****). Across all sequences, ‘first choices’ in the first trial of context 2 were more likely to object D than object B (Welch’s test, p = 0.0063) as well as more likely to object C than B (Welch’s test, p = 0.0049) (****Fig. 4B****). Comparison of choices in context 2 (first choice in first trial) with context 1 (first choice in last trial) showed that subjects not only chose C and D more likely than object B, but that they chose object D more likely than object C (Welch’s test, p = 0.0016) (****Fig. 4C****).These findings show that overall subjects used two different strategies in context 2 already in their first choices after the context change: a serial inference (choosing object C as first choice) and the re-indexing of object D as first choice at the 2^nd^ ordinal position. The direct comparison with context 1 suggests that the re-indexing strategy was more prevalent than the serial inference strategy (****Fig. 4C****). This pattern of result was found when considering all sequences (****Fig. 4A-C****), and also when considering only the subset of one-third of all sequences that were learned best in context 1 (with on average <5.33 errors per trial in context 1) (****Fig. 4D-F****). However, neither the pro-active re-indexing strategy, nor the serial inference strategy were evident when considering poorly performed sequences, i,e. when restricting the analysis to the subset of one-third of sequences that were performed poorly in context 1 (with on average >6.6 errors per trial in context 1) (****Fig. 4G-I****).“* (Result section, line 317)

__________________________________________________________________________
**Comment #40:** L212-215: Shouldn't this go in the previous paragraph, where you detail the strategies?

*Reply:* Here, we summarize the results and what they reveal about the choice strategies. To address the comment, we added clarifying sentences in the preceding paragraphs defining the different choice strategies more explicitly.

__________________________________________________________________________
**Comment #41:** L231 "Across all sequences": At this stage, I still don't understand if the sequences are the same series of objects for each individual, or if the objects and order change between each series. Because if the objects and order do not change, then one can simply argue that they have learned the orders of the objects for each context and just re-do them right after several sequences

*Reply:* We clarify in the revised text that 10-12 unique sequences (i.e. unique context 1+2 sequence pairs) were learned in each session at the end of the introduction section (please see reply to comment #34)

__________________________________________________________________________
**Comment #42:** L252: This info is very important and needs to come before. Also, you need to clarify your results section so that we understand over which dataset you perform each analysis

*Reply:* We adjusted the text and introduce this information earlier in the text by writing:

*“After the delayed match-to-sample task subjects performed an additional five to six context 1+2 sequence pairs.”* (Introduction section, line 168)

And that

*“Two or three of these late performed context 1+2 sequence pairs were the same as shown earlier in the session (i.e. they were repeated sequences), which allowed assessing longer-term memory effects.”* (Introduction section, line 148)

__________________________________________________________________________
**Comment #43:** L258: What is the point of the match-to-sample task here?

*Reply:* The match-to-sample task measures working term memory. Working memory has been suggested to be versatile mechanisms underlying sequence learning and the ability to manipulate learned sequences. We describe this more explicitly in the introduction and the discussion section by writing, e.g.

*“Computational work has shown that learning a sequence can be similarly achieved with long term memory that retains sequential relations in synaptic weights, or with working memory that retains sequential relations in recurrent network activity (Whittington et al., 2025). According to these insights the subject’s ability for to retain objects in working memory might be predictive of overall speed of learning sequences or the ability to flexibly swap objects in the sequence.”* (Discussion section, Line 408)

One strength of our study is that we were able to evaluate short term memory in the same session as assessing sequence learning. To our knowledge this has not been achieved so far in animal studies and is a novel aspect of our study.

__________________________________________________________________________
**Comment #44:** L261-262: What does it mean? You did not introduce what a "repeated sequence" is

*Reply:* We adjusted the text and now introduce this by writing

*“Two or three of these late performed context 1+2 sequence pairs were the same as shown earlier in the session (i.e. they were repeated sequences), which allowed assessing longer-term memory effects.”* (Introduction section, line 169)

__________________________________________________________________________
**Comment #45:** L295-296: I still don't understand what this does here. What do you match to what, for what?

*Reply:* We believe this comment refers to the neurophysiological findings that suggest that swapping (or in that study: retrieving 2-item sequences backwards) involves a short-term memory buffer. We expanded and re-wrote this section in the discussion section and write now:

*“… A neuronal correlate for such cue-triggered reconfiguration process has recently been suggested in an experiment that required NHPs to recall backwards the spatial order of 2- and 3-item sequences (Tian et al., 2024). Groups of frontal cortex neurons represented forward spatial 2-item sequences A-B until a cue instructed the subjects to recall the sequence backwards. During the re-coding of the forward into a backward sequence neuronal population activity in frontal cortex transiently encoded both, the forward A-B sequence and the swapped items A as a rank 2 object and B as a rank 1 object. This transient encoding of both, forward and backwards item order, was then followed by neuronal populations representing only the backward sequence that needed to be recalled (Tian et al., 2024). These findings suggest that swapping the order of adjacent objects involves a short-term memory that contains in separate neuronal populations the old item order (forward sequence) and the new item order (the backward item order). While these neuronal findings are limited to playing backwards adjacent items, we suggest that they provide a versatile framework to conceptualize the re-indexing of objects to non-adjacent positions in our study.”* (Discussion section, line 472)

If these neurophysiological findings are relevant for understanding behavior, then we expect working memory abilities will predict swapping of objects to different ordinal positions in a sequence.

__________________________________________________________________________
**Comment #46:** L297 "WM": Introduce this abbreviation before using it.

*Reply:* Thank you for finding this. The revised text avoids this abbreviation.

__________________________________________________________________________
**Comment #47:** L316-317 "the swapping occurred... error": But only after a few errors, no? They did not spontaneously change to the D without previous exposure to this task?

*Reply:* Swapping also occurred pro-actively, i.e. even without an error. We clarify this (also with additional Bayesian analysis of individual subjects), and adjusted the text which now refers to the specific relevant results figures. We write:

*“When a change in context indicated that non-adjacent objects B and D switched positions, three of four subjects swapped these objects more likely than erroneously choosing the next-ranked object C in the sequence (****Fig. 2B-F; 3****). The swapping occurred pro-actively, i.e. prior to making an error (****Fig. 4A-F; 5B****,****D,E****,****H****), as well as retro-actively, i.e. when subjects corrected an erroneous choice of B at the second ordinal position in context 2”* (Discussion section, line 438)

__________________________________________________________________________
**Comment #48:** L322-323 "for more sessions later in the experiment": This is confusing, it sounds as if something happening after influenced what happened before. Reformulate

*Reply:* Thank you for pointing us to this. We rewrote to:

*“… swapping became more likely over the course of the experiment when the swapping rule had been performed for more sessions (****Fig. 5H****).”* (Discussion section, line 455)

__________________________________________________________________________
**Comment 49:** L414 "stepping forward or reset of the slider position of the progress bar": Are you sure that monkeys actually use these cues? Please provide a reference. 

*Reply:* We did not intent to convey that the slider progress was needed to perform the task. It may have served as an additional motivational device. We clarify this aspect in the revised manuscript and now write

*“… the task paradigm provided immediate performance feedback of every choice in the form of the halo feedback and the stepping forward or backward of the slider position of the progress bar (****Fig. 1A****).While only the halo or only the slider progress would have been sufficient to provide feedback information this design aspect may have enhanced the likelihood subjects recognize when an object was chosen erroneously, which may facilitate learning.”* (Discussion section, line 590).

__________________________________________________________________________
**Comment #50:** L483-484: At this stage, it is still not clear what the difference between a sequence and a trial is

*Reply:* We added clarifying sentences at the end of the introduction section (please see reply to comment #34)

__________________________________________________________________________
**Comment #51:** L492: what is a sequence pair, is it context 1 + context 2?

*Reply:* Yes, this is correct and now explicitly defined at the end of the introduction section (please see reply to comment #34)

__________________________________________________________________________
**Comment #52:** L496-497: Why did the context vary in one condition but not in the other?

*Reply:* We motivate this variation in the results section by writing that we

*“...tested whether memory of the context background in which a sequence was learned influenced swapping behavior.”* (Results section, line 425).

We extended the results on this aspect and describe that

*“… [w]e found improved sequence learning performance on repeated versus early (initial) sequences when the context of the repeated sequence was the same compared to when the repeated sequence was shown on a different context background (Welch's t-test, p = 0.0083;* ***Fig. 6D****). However, this overall contextual facilitation did not modulate pro-active or retro-active swapping in context 2 (pro-active sapping: Welch's t-test: p = 0.3556;* ***Fig. 6E****; retro-active swapping: Welch's t-test: p = 0.1225;* ***Fig. 6F****).”* (Results section, line 428).

Because this is a “null result” we do not devote a discussion of the absence of an effect. The result means that the contexts did indeed help to recall the initial object sequence in context 1, but there was not an additional benefit on the swapping effect in context 2.

__________________________________________________________________________
**Comment #53:** L504-505: If they didn't pay attention to the features of the objects and still managed to solve the task, what would be the bias or the problem in the interpretation? Or in other words, why is that an important control?

*Reply:* We did not intend to convey that this was an important control. The distractor object could have been removed from the study without affecting the interpretation of any of the key findings. In the revised methods, we removed the sentence that conveyed that the distractor was “used to validate” that subjects paid attention to the features (Methods section, line 698).

__________________________________________________________________________
**Comment #54:** L509-519: It is still not clear what the point of this is. It is to ensure a time gap between the early and late sequences?

*Reply:* Working memory abilities could have correlated with sequence learning or with the swapping ability – and thereby constrain the interpretation on how subjects perform the task. We kindly refer to the reply to comment #44 and #46.

__________________________________________________________________________
**Comment #55:** L523: It is only now that I understand that a sequence is a set of objects in a specific order, and not a set of trials. Is that right? This needs to be explicated somewhere as it relies too much on the understanding of the reader and some parts of the text makes it confusing

*Reply:* We understand and more explicitly describe the design at the end of the introduction. We also refer to the Movie S1 at an earlier location. We were hoping that the movie would have been provided as a hyperlink to the reviewers (and will be provided as a link to the reader) to accompany the design shown in Figure 1A-B, because the movie might disambiguate how the task is performed. We kindly refer to the replies to comments #30-32 that aim to address clarity of the design.

__________________________________________________________________________
**Comment #56:** L548 formula: What are a, b and c here? 

*Reply:* We clarify the formula by revising the text to:

*“…we fitted an exponential decay function … of the form (*$a*\exp\left( -b*x \right)+c$*), where a represents the amplitude of decay, b represents the speed of decay (plotted in* ***Suppl. Fig. 1E****), and c represents the asymptotic baseline. The same decay function was applied to each session individually, and the speed of decay was plotted to compare the rate at which different error types diminished.” (Methods section, line 751).*

__________________________________________________________________________
**Comment #57:** Figures: make sure that they are adapted to the vision of colour-blind people.

*Reply:* We addressed this comment by reviewing and updating our color palette choices using a color-blindness simulation tool (<https://davidmathlogic.com/colorblind/#%23D81B60-%231E88E5-%23FF8F07-%23004D40> ) to ensure that all figure elements are distinguishable for individuals with various types of color vision deficiencies. Specifically, Figure 2D was updated with a brighter color palette to improve visibility, and Figure 2E now uses a dashed line versus a solid line to help differentiate between magenta and gray, which can be challenging for readers with protanopia. We are committed to making our figures accessible to all readers and have applied these principles consistently throughout the manuscript.

__________________________________________________________________________
**Comment #58:** Fig 1C : in context 1, 2 or both? It sounds from the legend of Fig1 as if this is only in context 1. How do you explain that they learn A very easily and then it dramatically drops for B?

*Reply:* We believe object A is performed so well because of a primacy effect, i.e. it does not need knowledge of a sequential position. Performing well on later objects require an understanding of the sequence position of the object.

We added explicitly the information that Figure 1C uses behavioral from sequences in context 1.

__________________________________________________________________________
**Comment #59:** Fig 1D: "for at least the next 3 trials" is this a new criteria? Please unify across the manuscript.

*Reply:* We ensured in the revision that we used a unified, identical learning criteria across all analysis.

__________________________________________________________________________
**Comment #60:** Fig 2F and 2G: it is not clear whether the "first choices in a trial prior..." graph belongs to F or G, maybe redesign this figure or give another letter to this graph to make it clearer.

*Reply:* Thank you for suggesting this - We added a letter to the difference plot and explicitly refer to that letter in the legend and text. These are now panels Figure 2E and 2F in the revised manuscript (we removed original figure panels 2B+2C in response to another reviewer comment).

__________________________________________________________________________
**Comment #61:** Fig 3J: this graph is hard to process, can you explicit it more, directly on the figure or/and in the legend?

*Reply:* We expanded the description in the figure legend to:

*“Trial-by-trial differences of the ‘first choice’ choice probabilities after subjects chose correctly object A context 2. The blue line shows the difference of the likelihood to choose D rather than B (‘D-B’), while the other lines show the difference of the likelihood to choose C rather than B (grey: ‘C-B’) and to choose rather than C (yellow: ‘D-C’). Object B was less likely chosen than C and D as first choice in the first trial of context 2, and object C was less likely chosen than D starting from the 7th trial.”* (Figure 4J, line 1040)

__________________________________________________________________________

**Comment #62:** Fig 4: Can you make it clearer, on the y-axes, in which direction the preferences are going? For example, in Fig4B, "D vs B": is the preference for D below or above zero?

*Reply:* We added this detail in the figure legend Fig 5B and 5C and write:

*“(****B****) Pro-active swapping in context 2 (values above 0 reflect that D is more likely being chosen than B after choosing A): Anticipating that D is in 2^nd^ position in context 2 is similarly evident in all conditions. (****C****) Retro-active swapping after erroneously choosing object B in context 2 (values above 0 reflects that D is more likely being chosen than C after choosing B): Difference of choosing object D vs. C.”* (Figure 5 legend, line 1053).
